# Heterogeneous Fenton Reaction Enabled Selective Colon Cancerous Cell Treatment

**DOI:** 10.1038/s41598-018-34499-0

**Published:** 2018-11-08

**Authors:** Kuan-Ting Lee, Yu-Jen Lu, Shao-Chieh Chiu, Wen-Chi Chang, Er-Yuan Chuang, Shih-Yuan Lu

**Affiliations:** 1 Department of Chemical Engineering, National Tsing Hua University, Hsinchu, 30013 Taiwan Republic of China; 2 Department of Neurosurgery, Chang Gung Memorial Hospital, Taoyuan, 33302 Taiwan Republic of China; 3 Center for Advanced Molecular Imaging and Translation, Chang Gung Memorial Hospital, Taoyuan, 33302 Taiwan Republic of China; 4 Graduate Institute of Biomedical Materials and Tissue Engineering, Taipei Medical University. College of Biomedical Engineering, International PhD program of Biomedical Engineering and Translational Therapies, Taipei, 11042 Taiwan Republic of China

## Abstract

A selective colon cancer cell therapy was effectively achieved with catalase-mediated intra-cellular heterogeneous Fenton reactions triggered by cellular uptake of SnFe_2_O_4_ nanocrystals. The treatment was proven effective for eradicating colon cancer cells, whereas was benign to normal colon cells, thus effectively realizing the selective colon cancer cell therapeutics. Cancer cells possess much higher innate hydrogen peroxide (H_2_O_2_) but much lower catalase levels than normal cells. Catalase, an effective H_2_O_2_ scavenger, prevented attacks on cells by reactive oxygen species induced from H_2_O_2_. The above intrinsic difference between cancer and normal cells was utilized to achieve selective colon cancer cell eradication through endocytosing efficient heterogeneous Fenton catalysts to trigger the formation of highly reactive oxygen species from H_2_O_2_. In this paper, SnFe_2_O_4_ nanocrystals, a newly noted outstanding paramagnetic heterogeneous Fenton catalyst, have been verified an effective selective colon cancerous cell treatment reagent of satisfactory blood compatibility.

## Introduction

An ideal cancer treatment should exclusively target cancer cells without damaging normal cells. However, in practice, this is quite challenging. Tumor biology has elucidated that cancerous cells are characterized with high intrinsic oxidative stresses. Compared to normal health cells, most cancerous cells contain much higher levels of hydrogen peroxide^1^. Some studies reported that greatly elevated H_2_O_2_ levels were detected in cancerous cells compared to normal cells because of the enhanced metabolic rate and the rapid proliferation of cancer cells^2^. These high levels of H_2_O_2_ in cancer cells have been utilized to design novel therapeutic approaches for killing cancer cells^3^. Heterogeneous Fenton reactions, originally developed for catalytic degradation of organic pollutants^4^, produce highly reactive hydroxyl free radicals *via* redox reactions between solid state iron-containing catalysts (crystal ferric ions) and absorbed H_2_O_2_ molecules^5^. The heterogeneous Fenton reactions can efficiently produce hydroxyl free radicals, particularly in environment with high concentrations of H_2_O_2_, e.g., cancer cells. Cancer cell eradication can thus be achieved through endocytosing efficient heterogeneous Fenton catalysts into cancer cells to trigger the generation of highly reactive hydroxyl radicals. A mechanism, however, must exist to protect normal cells from possible attacks by hydroxyl radicals when the treatment is applied.

Catalase, an antioxidative enzyme abundant in normal cells, can catalyze the decomposition of hydrogen peroxide into oxygen and water with an extremely high efficiency. Cancerous cell, on the other hand, are quickly growthing cells that acquire elevated H_2_O_2_ levels and possess a negligible amount of catalase compared to normal cells^6^. During the treatment with heterogeneous Fenton reactions, triggered by endocytosing Fenton catalysts, catalase at normal physiological levels can protect normal cells by effectively suppressing the formation of hydroxyl radicals^7^. Nevertheless, cancer cells, which possess a limited amount of catalase but a high level of H_2_O_2_, are attacked by the generated hydroxyl radicals and thus eradicated^8^.

Colorectal cancer is a cause of morbidity with mortality in human population. Earlier research shows that, in colorectal cancer development, the active level of catalase is reduced^9^. In our previous investigation, SnFe_2_O_4_ nanocrystals (NCs) have been proven effective for treating lung cancer cells^10^. Here, we explore their eradication efficacy toward colon cancer cells with deeper insights derived from relevant biomedical characterizations. For instance, this iron based paramagnetic nanomaterial may exhibit strong contrasts in MRI imaging, one of the most powerful diagnostic tools in medicine. In addition, the blood compatibility of the functional nanomaterial is a vital prerequisite for its usage in bio-imaging, drug delivery system, and gene treatment.

In this study, these SnFe_2_O_4_ NCs were used for the selective treatment of colon cancerous cells. The SnFe_2_O_4_ NCs were farbricated through a single-step carrier solvent assisted interfacial chemical reaction procedure^11^. These SnFe_2_O_4_ NCs went through a certain extent of aggregation when dispersed in saline for cell treatment applications, depending on whether or not sonication was applied and the concentration of the suspension^12^. First, the effect of the size of the SnFe_2_O_4_ aggregates on the treatment efficacy was investigated. As expected, smaller-sized SnFe_2_O_4_ aggregates, obtained from sonication treatment at an appropriate suspension concentration, were advantageous in cellular internalization of the SnFe_2_O_4_ nano-agregates and following yielding of hydroxyl radicals *via* heterogeneous Fenton reactions. The successful cellular internalization of the SnFe_2_O_4_ aggregates into cells, has been proven with confocal laser scanning microscopy (CLSM) previously, and the paramagnetic property of the SnFe_2_O_4_ aggregates was elucidated with a superconducting quantum interference device (SQUID) and magnetic resonance imaging (MRI) technique^13,14^. The blood compatibility of the SnFe_2_O_4_ aggregates was also studied. Furthermore, the concentrations of the hydroxyl free radical and catalase in both normal and colon cancer cells were quantified with an fluorescent staining method^15^, confirming the proposed characteristic differences between normal and cancer cells in terms of H_2_O_2_ and catalase concentrations as described above. Finally, the efficacy of the SnFe_2_O_4_ NC-triggered heterogeneous Fenton reaction cell treatment was confirmed with cell viability measurements. The treatment was proven to be effective at eradicating colon cancer cells, whereas was benign to normal colon cells, thereby extending this selective therapy to colon cancer cell eradication.

## Results and Discussion

Low levels of catalase activity were characterized in most cancer cells including the colon cancer samples examined. These cancer cell samples were thus more vulnerable to oxidative stresses induced by ROS-generating reagents. Thus, elevating ROS levels provides a rational means to abolish cancer cells, without appreciably damaging normal cells because of the presence of high levels of endogenous catalase in normal cells. Much research effort has been focused on developing strategies aiming at creating cytotoxic oxidative stresses for cancer therapy^16–18^.

The heterogeneous Fenton reaction is a critical reaction in which the lattice ferric ions of the solid-state Fenton catalyst convert hydrogen peroxide into very toxic hydroxyl free radicals that raise ROS stresses for colon cancer cell eradication. The present study is to apply SnFe_2_O_4_ NCs in the bio-pharmacological field and investigate, using a fluorescent imaging approach, their *in vitro* efficacy in producing ROS, paramagnetic property, blood compatibility, and subsequent cytotoxicity toward colon cancer cells.

### Characteristics of SnFe_2_O_4_ aggregates

In the bio-pharmacological field, major studies have highlighted the importance of controlling the particle size, shape, and chemistry for drug delivery efficiency. In many cases, agglomeration/aggregation among solid particles is caused by prevailing attractive forces (van der Waals). Physical breakup, for example sonication, was identified as being a convenient way to achieve mechanical separation to lessen the extent of aggregation/agglomeration in the suspensions^19^. Besides, elevated particulate densities in solution tend to favor serious aggregation/agglomeration. Therefore, it is essential to adjust the concentration of the particulates and to apply sonication when preparing the SnFe_2_O_4_ NC suspensions in physiological fluids.

Heterogeneous Fenton reactions make hydroxyl radicals *via* redox reactions on the surface lattice ferric ions of the solid-state catalys and absorption H_2_O_2_ molecules^20^. It is therefore expected that SnFe_2_O_4_ aggregates of large reactive surface areas will efficiently produce hydroxyl radicals in colon cancer cells. A convenient bio-probe as marker of intracellular reactive oxygen species is 2,7-dichlorodihydrofluorescein diacetate (DCFH-DA). It is a good indicator for hydroxyl radicals but is insensitive toward H_2_O_2_^21–23^. Additionally, intracellularly endogenous catalase can efficiently scavenge hydrogen peroxide^24^, consequently suppressing the production of cytotoxic hydroxyl radicals.

**Figure 1:**
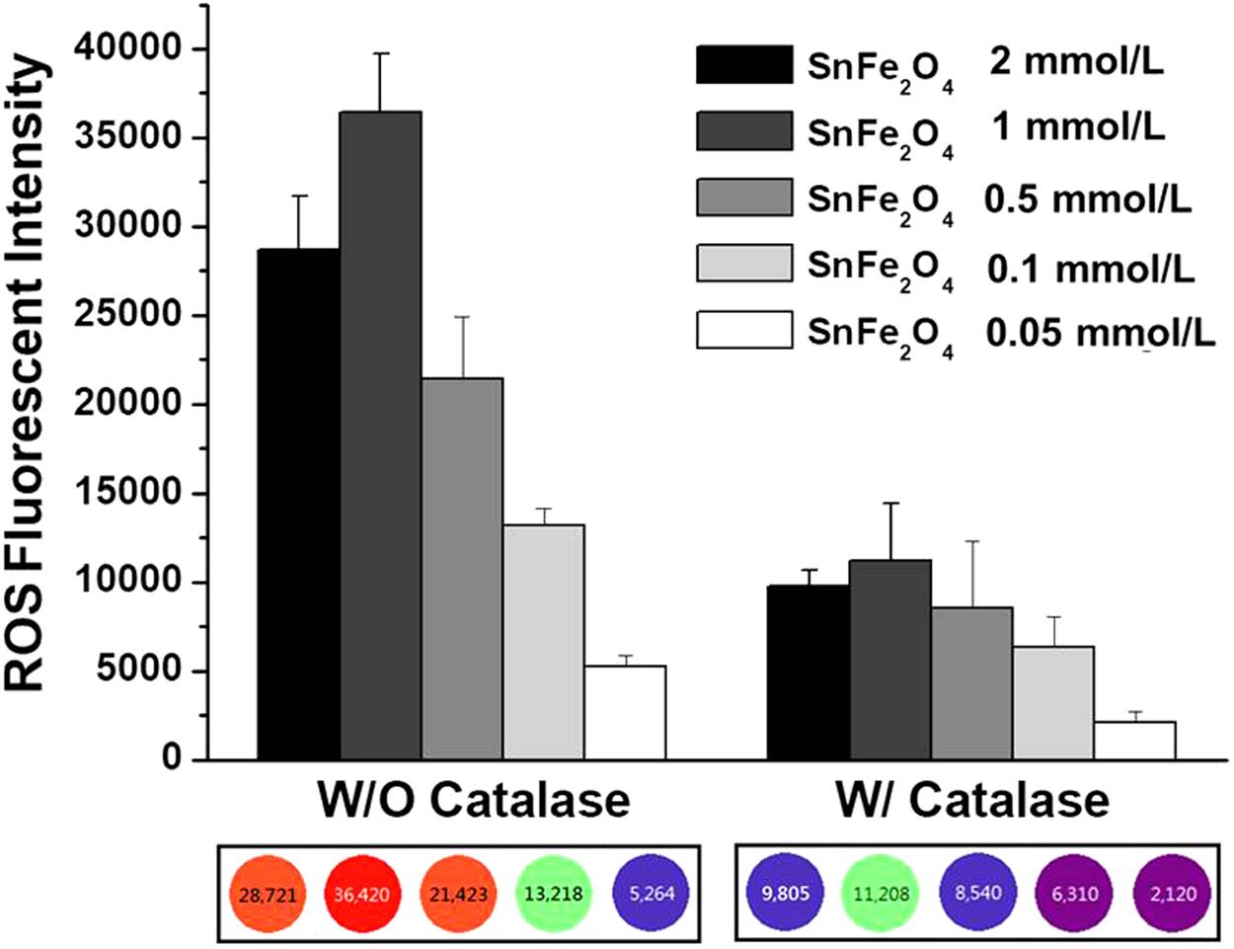
Levels of ROS generated by heterogeneous Fenton reactions between SnFe_2_O_4_ aggregates and H_2_O_2_ (500 mM) at increasing SnFe_2_O_4_ concentrations (0.05, 0.1, 0.5, 1, and 2 mmol/L) in the presence or absence of catalase, as determined by microplate reader.

As shown in Fig. 1, for the without catlase case, the amount of hydroxyl radicals created was positively correlated with the concentration of the SnFe_2_O_4_ aggregate, at 0.05~1 mmol/L in the presence of H_2_O_2_ (500 mM) and with application of sonication. This was expected since more SnFe_2_O_4_ was available to generate ROS such as hydroxyl radicals with an increasing SnFe_2_O_4_ agrregate concentration. Nevertheless, once the concentration of the SnFe_2_O_4_ aggregates was further increased to reach 2 mmol/L, the catalytic efficiency of the hydroxyl radical generation decreased.

We speculated that in this case, the effective catalytic surface area of the SnFe_2_O_4_ agrregates had in fact diminished because of the severe aggregation of the SnFe_2_O_4_ NCs. It was also interesting to note the lack of hydroxyl radicals when there was present of catalase with SnFe_2_O_4_. This confirms that the source of the hydroxyl radicals was the catalytic conversion of H_2_O_2_ by SnFe_2_O_4_.

**Figure 2:**
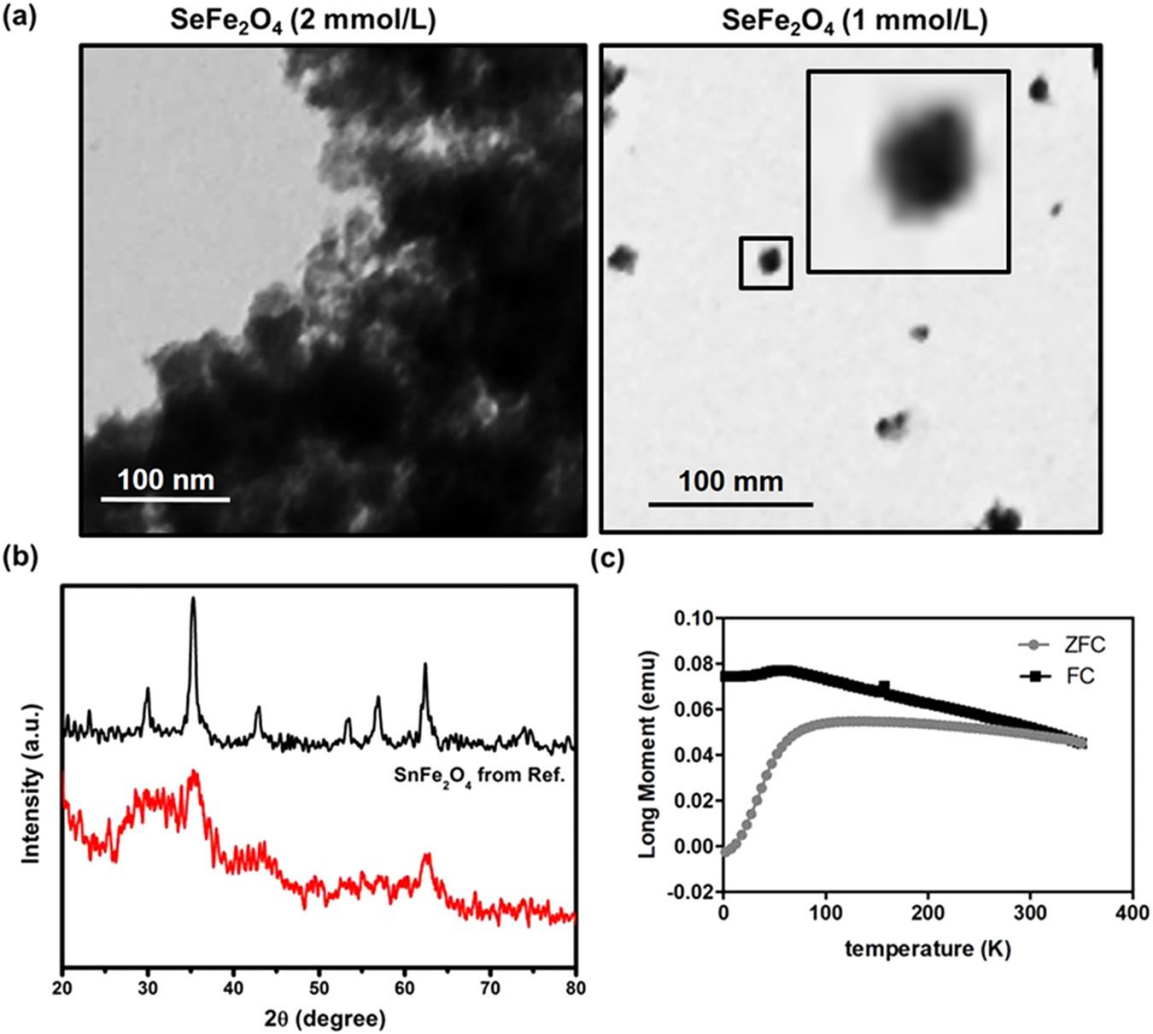
(**a**) TEM images of SnFe_2_O_4_ aggregates formed at concentrations of 2 and 1 mmol/L. (**b**) XRD patterns of present SnFe_2_O_4_ aggregates and Ref. (**c**) Long moment vs. temperature curve of SnFe_2_O_4_ NCs_._

Figure 2a shows the TEM image of the SnFe_2_O_4_ aggregates obtained under sonication for 1 h at 37 °C at two particulate concentrations of 1 and 2 mmol/L. It is evident that the SnFe_2_O_4_ NCs went through aggregation process in physiological saline solution owing to the decrease of the electrostatically repulsive interactions caused by the presence of free counter-ions of Cl^−^ and Na^+^ offered by the solution of saline. Aggregate sizes around below 20 nm, however, were obtained at 1 mmol/L, much smaller than those obtained at 2 mmol/L, which were micron-sized. The data verify our conjecture for the decreased hydroxyl radical level at 2 mmol/L as compared to that at 1 mmol/L. Higher particle concentrations cause more severe aggregation, resulting in decreases in the effectively exposed catalytic surface areas for generating hydroxyl radicals from H_2_O_2_. According to a previously published article, smaller particulates enable greater intra-cellular internalization compared to larger ones, and thus smaller particulates can be more effectively uptaked by the cells^25^. The SnFe_2_O_4_ aggregates of below 20 nm obtained at the suspension concentration of 1 mmol/L were used for subsequent studies. It is expected that these SnFe_2_O_4_ aggregates can be readily internalized into colon cells, normal or cancerous, and produce large amounts of hydroxyl radicals in colon cancer cells to significantly raise the ROS stresses to kill the colon cells. Here, the crystalline structure of these SnFe_2_O_4_ aggregates was studied with XRD. As shown in Fig. 2b, the diffraction pattern of the SnFe_2_O_4_ aggregates is in good match with that of the SnFe_2_O_4_ nanocrystals of ref.^9^, confirming the composition of the catalyst to be SnFe_2_O_4_. Furthermore, SnFe_2_O_4_ is a paramagnetic material, responsive to externally applied magnetic fields, and its paramagnetism was verified with the long moment vs. temperature curve presented in Fig. 2c, which was measured with a SQUID magnetometer.

Bio-functional traits and cellular uptakes of particulates in active substance delivery are highly dependent on the geometrical and structural features, such as size and shape, of the particulates^26,27^. Typically, particles with spherical morphology can be more swiftly internalized by the cells than particles with irregular shape^28^. The particle size was also found to be associated with the cell internalization behavior and their endocytic pathway, crucially dictating the intracellular fate and consequent biologic effects of the particles. It has been shown that particles with a dimension of below 500 nm could accomplish significantly higher cellular uptake than could larger particles^29^. We have verified that the present SnFe_2_O_4_ aggregates were successfully internalized by living cells^10^. As proposed in literature, cellular uptakes of nanoparticles of sizes below 200 nm would be observed to involve specific clathrin-coated pits^30^. In a physiological environment, the metal oxide materials taken in can be gradually degraded within the lysosomal space and are eventually converted into free metal ions that could be rapidly urinated *via* bladder. In practical circumstances, this designed formulation could be applied to carry out an *in vivo* study through an intravenously administrated route for colon cancer treatments, in which the SnFe_2_O_4_ aggregates accumulate within the colon cancer cells through either the retention (EPR) effects and enhanced permeability^31^ or magnetically guided drug targeting (MGDT)method^32^.

### MRI evaluation

MRI has been considered a useful medical imaging technique in radiology and physiological processes for the anatomy of the human body^33^. MRI scanners operate radio waves, robust magnetic fields, and field gradients to generate living images of the body. Furthermore, magnetic particle imaging (MPI) has been considered a novel imaging modality using paramagnetic iron based particles as a substance of tracer. This newfangled tomography of radiation-free imaging technique offers quick, sensitive, background-free, straight quantifiable 4 dimensional (4D) reports concerning the spatial distribution of the magnetic substance at very high temporal resolutions, ultra-sensitivity, and excellent spatial resolutions.

**Figure 3:**
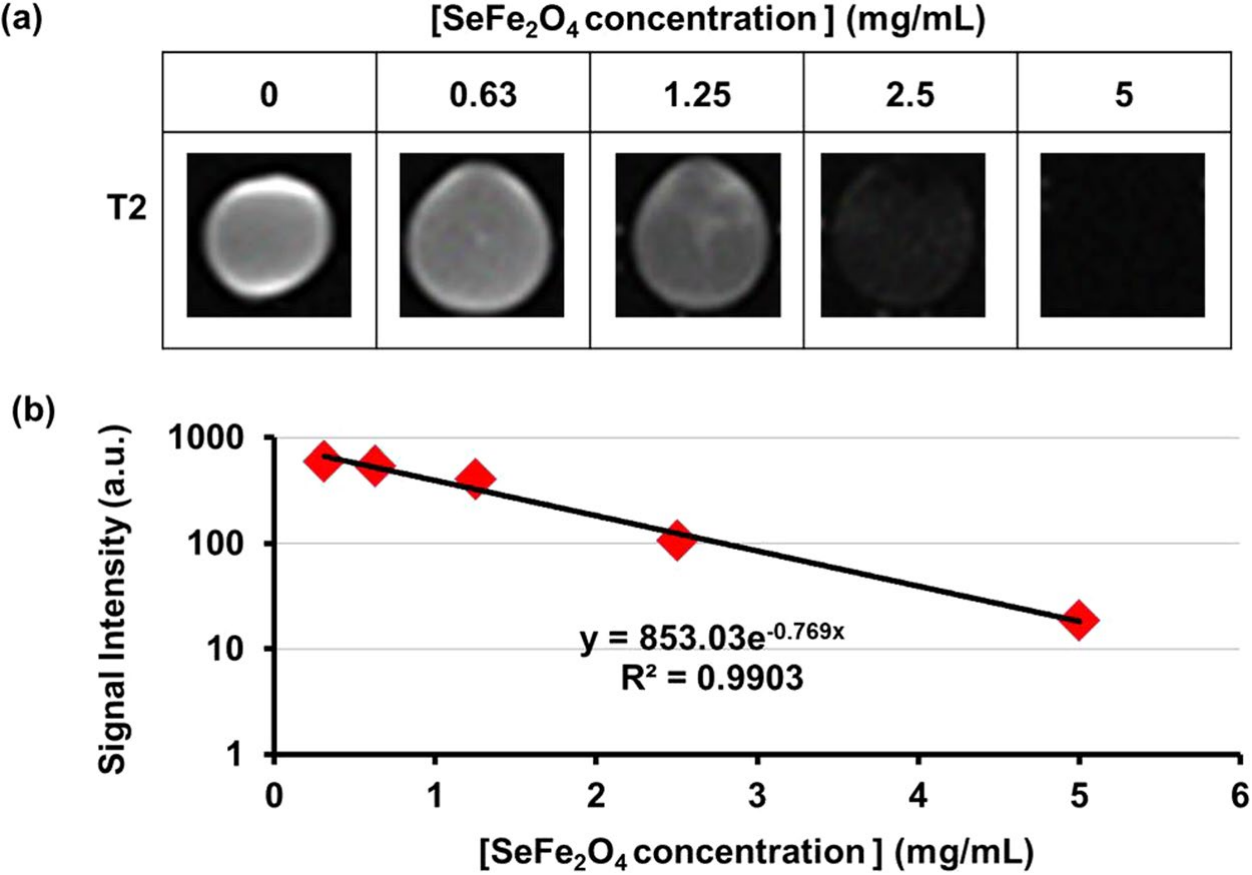
T2-weighted MR images of SnFe_2_O_4_ (**a**) of increasing concentration and (**b**) the quantitative data.

MRI enables sensitive and specific detection of (para)magnetic nano-carriers in biological systems^34^. Here, it was applied to quantify the SnFe_2_O_4_ aggregates. To evaluate their T2-enhancing capability, SnFe_2_O_4_ aggregate suspensions of increasing concentrations were examined by T2-weighted MRI. The acquired outcomes suggested that the paramagnetism of the SnFe_2_O_4_ aggregates was promptly detectable by MRI. Among increasing amounts of the SnFe_2_O_4_ suspensions, the signal intensity of MRI decreased (Fig. 3a,b). As well known, MR imaging is a non-invasive approach that has become the most vital noninvasive diagnostic means in many medical applications. MRI not only provides excellent morphological information but also possesses the ability to provide the best soft tissue contrast compared to all techniques of clinical imaging.

### Immunofluorescence of DCFH-DA

**Figure 4:**
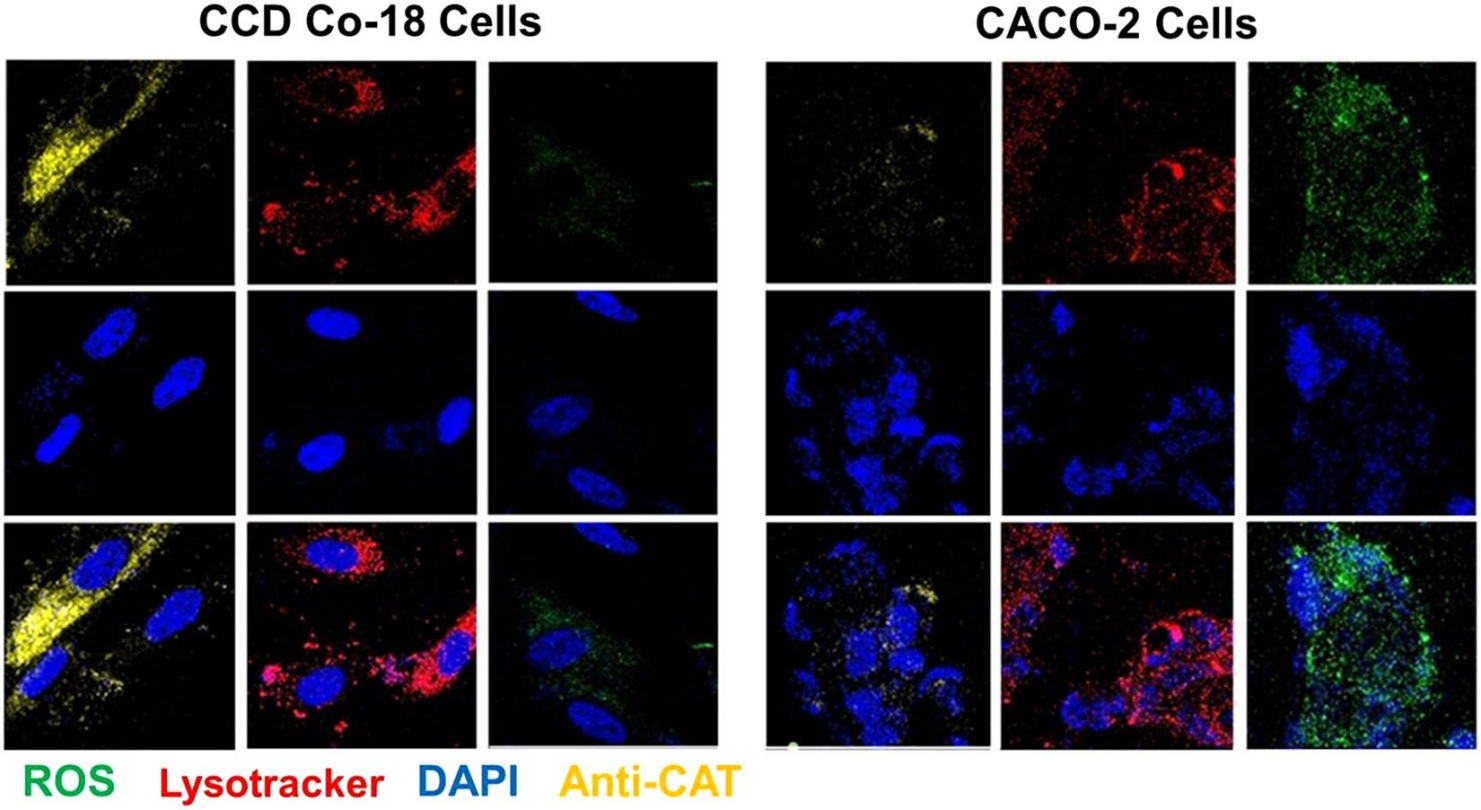
CLSM results concerning intracellular interaction of SnFe_2_O_4_ aggregates in caco-2 (colon cancer cells) or CCD Co-18 (normal human colonic fibroblast) that were imaged 12 h after treatment. Red, blue, green, and yellow colors represent signals of LysoTracker, DAPI, ROS, and anti-CAT, respectively.

The majority of colon cancerous cells in general possess extraordinarily few anti-oxidative bio-enzymes. Interestingly, the levels of intracellularly endogenous catalase in healthy normal colon cells are meaningfully greater than those examined through confocal in cancer cells (Fig. 4). One possible explanation for the observed outcomes may be that catalase may affect at either the protein level or mRNA throughout the progressing period of the cancerous cells^35^. Consequently, the intracellularly endogenous catalase can essentially diminish the amount of H_2_O_2_ existing in normal cells by decomposing H_2_O_2_ into oxygen (O_2_) and water (H_2_O).

Owing to the presence of an enough amount of intracellular catalase, despite the cellular internalization of the SnFe_2_O_4_ aggregates, the normal cell group treated with the SnFe_2_O_4_ aggregates was incapable of converting enough H_2_O_2_ into hydroxyl radicals, as observed from the fluorescent image results (Fig. 4). The yielding of hydroxyl radicals by the SnFe_2_O_4_ aggregates was suppressed in normal cells because of the presence of sufficient amounts of catalase, which was at considerably greater concentrations in normal colon cells than in colon cancerous cells. In addition, it has been recognized that a catalase protein is capable of decomposing millions of H_2_O_2_ molecules into oxygen (O_2_) and water (H_2_O) in short period (one second). Aiming at cancer cells, the SnFe_2_O_4_ aggregates could convert excessive levels of intracellular hydrogen peroxide into a considerable level of ROS which possibly is mainly hydroxyl radicals.

An important impact of this designed method is inhibiting heterogeneous Fenton reaction by using catalase *via* disintegration of hydrogen peroxide. In addition, it is acknowledged that the expression of catalase in normal cells has been considered as mediator at the protein, polypeptide, delivering message, and bio-actively molecular levels. The cancerous cells applied in this study have minimal catalase active levels^36^. Swiftly actively growing cells, for instance cancerous cells, make aberrantly large amounts of hydrogen peroxide (H_2_O_2_). This would enhance the oxidative stresses experiencing transformation of neoplastic and consequently improve the therapeutically targeting of cancerous cells through differences in levels of catalase.

### Hemolysis Study

**Figure 5:**
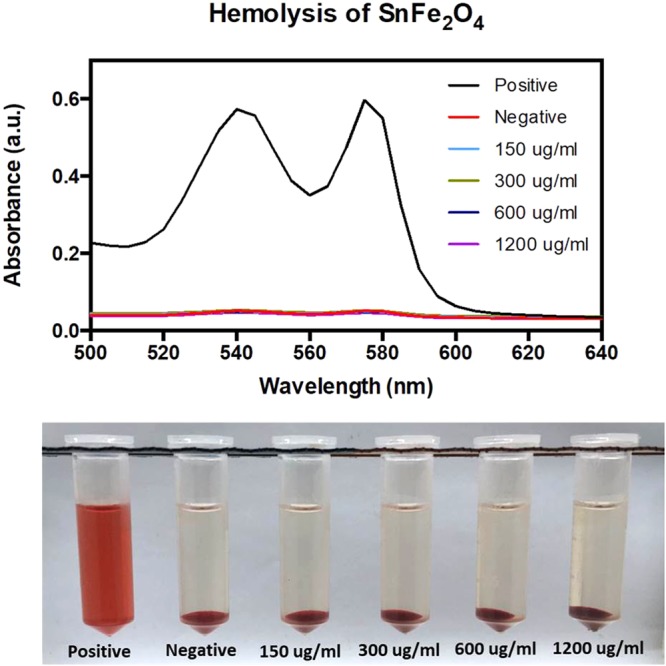
Hemolysis study results of test samples at different SnFe_2_O_4_ concentrations.

The hemolysis (destructing red blood cells) *in vivo* would be associated with jaundice, anemia, or other undesired pathological circumstances, thus the hemolytic potential of all pharmaceuticals of intravenous administration should be estimated. Drug carrier system and nanomaterial-based devices are emerging as replacements to traditional therapeutic drugs, and *in vitro* test of their biocompatibility with blood substances is an essential part of the primary pre-clinical development. The unique physicochemical properties of nanomaterials may lead to bio-interactions with erythrocytes to differ from those detected for traditional pharmaceuticals, and may also lead to interfering with regulated *in vitro* tests. The results of the test samples with different amounts of SnFe_2_O_4_ incubated with harvested red blood cells from rats suggested that no destructed red blood cells were observed (Fig. 5). However, the red blood cell is placed in pure distilled water (a hypotonic solution) in which the water molecules are in a high concentration external to the red blood cell and water can thus move into the red blood cell, causing rupture possibly due to the different osmotic pressure.

### Cytotoxicity

For producing an effect of cytotoxicity, hydroxyl radicals would destroy the DNA backbone of sugar phosphate by receiving hydrogen (H atoms) from deoxyribose and then damaging bases of DNA by the addition of generated OH onto the double bonds of the purine ring. Once DNA has noted to be disturbed by these harmful hydroxyl radicals, this reacting procedure has to be involved in the close DNA vicinity^37^. As is well recognized, hydroxyl radicals (OH) are expressively more energetic and therefore much more toxically offensive than H_2_O_2_^38^.

**Figure 6:**
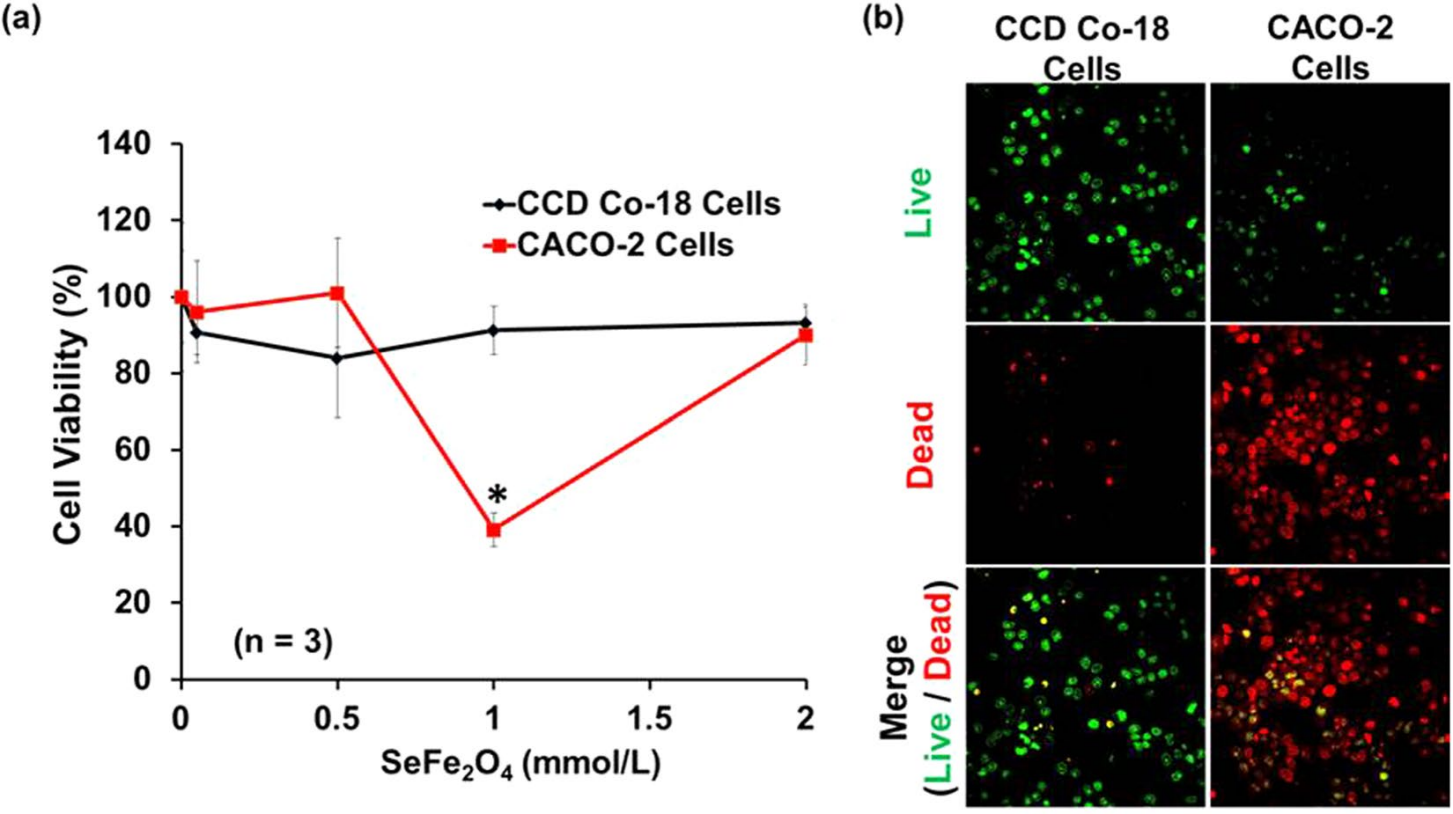
(**a**) Quantitative results obtained from MTT assay. ^*^Statistical significance indicated by *p* < 0.05. (**b**) Corresponding fluorescent images of tested cell co-stained with LIVE(green)/DEAD(red)® viability/cytotoxicity assay kit for human colon cancer and normal conlon cells (1 mmol/L).

As evident from Fig. 6, normal colon cells survived well in the treatment of the SnFe_2_O_4_ aggregates, showing high cell viability. This was owing to the presence of high catalase levels, notably suppressing death of apoptotic cell induced by the SnFe_2_O_4_ aggregates. On the contrary, the SnFe_2_O_4_ aggregates imposed a pronounced cytotoxic bio-action in colon cancer cells (Fig. 6). These fluorescently imaged observations revealed that heterogeneous based Fenton reactions, bio-performing *via* the prepared SnFe_2_O_4_ nano-aggregates, greatly intensify the amount of ROS for initiating damage of colon cancer cells. These SnFe_2_O_4_ aggregates, however, has been considered as safe toward normal colon cells. The corresponding quantitative cellular viability outcomes were examined and are shown in Fig. 6a,b. The group of colon cancer cells displayed a drastic (*p* < 0.05, 1 mmol/L) decrease in cell viability, suggesting the apparent cytotoxic effect of the SnFe_2_O_4_ aggregates of a proper dimension for endocytosis toward colon cancer cells.

The high concentration suspensions (group of caco-2 cell, 2 mmol/L) will experience severe particle aggregation as shown in the TEM graph, which limits efficacy of the cellular uptake due to the huge particle dimension. This observed phenomenon was consistent with the MTT cytotoxicity results. As well known, this metal based SnFe_2_O_4_ aggregates are likely transported away through the compartment of vessel as dissolved metal oxide ionic species and then cleaned through the bladder and kidneys. Accordingly, this prepared SnFe_2_O_4_ aggregates should very feasibly to be both drug delivery carrier system and a therapeutically active substance for H_2_O_2_-rich aims, for instance cancerous cell microenvironments.

## Conclusions

**Figure 7:**
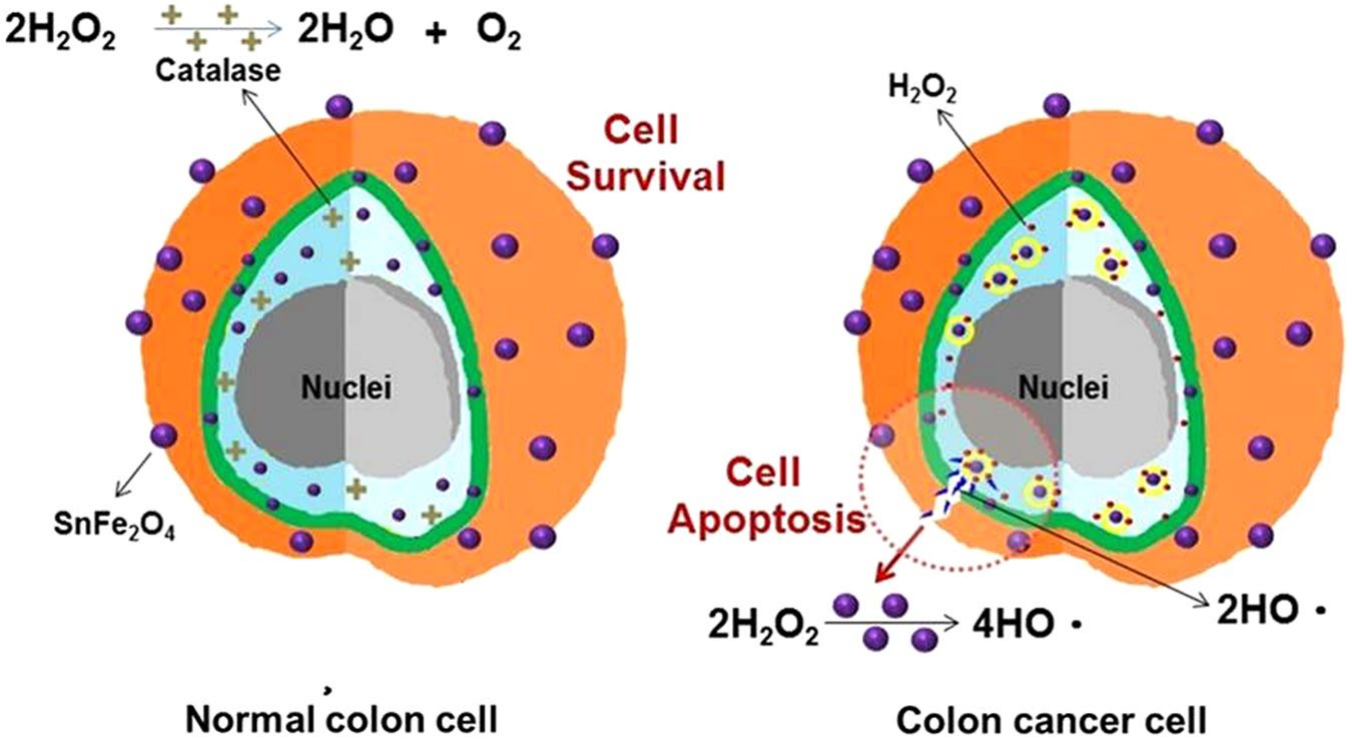
Schematic illustration shows that SnFe_2_O_4_ nanocrystals, a recently noted outstanding magnetically guidable heterogeneous Fenton catalyst, were proven to be effective in selective treating of colon cancer cells.

A H_2_O_2_-specific redox reagent of SnFe_2_O_4_ NCs was well developed to function as a novel therapeutic substance for selective therapy toward colon cancerous cells. Our current data proved that nano-dimension SnFe_2_O_4_ considerably enhance intracellular ROS hydroxyl radical generation through heterogeneous type Fenton reactions for endogenous H_2_O_2_, ultimately forming a noticeable effect of toxicity against colon cancerous cells, as theorized in Fig. 7. On the contrary, normal colon cells were secured by high levels of catalase which scavenges the hydrogen peroxide molecules before the heterogeneous Fenton reaction should proceed to generate hydroxyl radicals, as illustrated in Fig. 7. These consequences validate the efficacy of the developed SnFe_2_O_4_ aggregates for anti-colon cancer treatment. This developed SnFe_2_O_4_ NCs are paramagnetic, verified by MRI and SQUID experiments, and have blood compatibility. In the future, further studies are on-going to explore the physiological interactions between the SnFe_2_O_4_ aggregates and real organs for acquiring bio-information on the *in vivo* anti-tumor efficacy, biodistribution, *in vivo* toxicology, and excretion rate form kidneys.

## Methods

### Materials

All reagents and chemicals used were of analytical grade and were obtained from Sigma-Aldrich (St. Louis, MO, USA) unless otherwise stated. Reagents for cell study were purchased from Life Technologies (Carlsbad, CA, USA).

### Preparation of SnFe_2_O_4_ nanocrystals (NCs)

SnFe_2_O_4_ NCs were prepared with a single-step carrier solvent assisted interfacial chemical reaction method^11^. In brief, a precursor aqueous solution was first formulated by dissolving a suitable amount (0.0469 M) of SnCl_2_ (98%, obtained from Alfa Aesar) in 62.5 mL ethanol (Sigma-Aldrich, 99%), followed by adding a stoichiometric amount of Fe(NO_3_)_3˙_9H_2_O (98%, J.T. Baker). An amount of 62.5 mL of the precursor solution was supplemented into chloroform (99%, Sigma) in a same volume, to produce the organic phase of the liquid-liquid interfacial reaction enviroment. The aqueous phase solution was made by dissolving a desired amount (1 M) of NaOH (Showa, >95%) in 125 mL of distilled (DI) water, and was next dripped slowly to the organic phase to generate the liquid-liquid interfacial reaction system with the aqueous domain floating on top of the organic phase. The interfacial reaction performed at room temperature for 60 min under magnetic stirring in the organic phase. The product SnFe_2_O_4_ NCs were alternately washed with distilled (DI) water and ethanol several times for removing impurities and were then collected with a centrifuge (8000 rpm, RT, 10 min). They were further dried in an oven (80 °C) overnight for later use.

### Characterization of SnFe_2_O_4_ NCs

The SnFe_2_O_4_ NCs were added to physiological fluids for subsequent experiments. These NCs, however, aggregated in the aqueous phase due to a lack of strong electrostatic repulsive forces between the NCs, caused by the presence of the counter-ions supplied by saline, leading to aggregation dominated by the attractive van der Waals forces. The extent of NC aggregation and thus the aggregate size were controlled by the application of sonication and adjusting the suspension concentration. Sonication was applied using a sonicator with a microtip probe (JY92IIN, China).

Basically, the smaller nanosizes of the SnFe_2_O_4_ aggregates, acquired from sonicated NC suspensions of appropriate concentrations, can offer larger surface active areas for acquiring higher efficiencies in converting H_2_O_2_ into toxic hydroxyl radicals. To confirm this hypothesis, a DCFH-DA tracking technique was used to record the level of the H_2_O_2_-derived hydroxyl radicals. Briefly, 1 mL of the SnFe_2_O_4_ NC suspensions of given concentrations (0.05, 0.1, 0.5, 1, and 2 mmol/L) was mixed with H_2_O_2_ (500 mM) and DCFH-DA (at 80 μM) for 20 min at 37 °C in the dark. Fluorescent signal was monitored on a microplate reader. Quantifiable data were displayed as the fluorescent intensity. Catalase, an effective scavenger of H_2_O_2_, is anticipated to efficiently deplete H_2_O_2_ thus to reduce the concentration of the hydroxyl free radicals. To examine the influence of catalase on the level of hydroxyl radicals of the samples, the absence or presence of catalase was selected as an experimental parameter. The morphological change and dimension of the SnFe_2_O_4_ aggregates were observed and imaged with transmission electron microscopy (TEM) (Hitachi H-600, Tokyo, Japan). The X-ray diffraction (XRD) and the superconducting quantum interference device (SQUID) study were also performed to characterize the crystalline structure and paramagnetism of the SnFe_2_O_4_ aggregates.

### Cell study

A human colon cancer cell line, caco-2, acquired from the Bioresource Collection and Research Center (BCRC 60182), was maintained and cultured in Eagle’s media added with 1% streptomycin/penicilin, 1% 1-glutamine with 20% fetal bovine serum (FBS) at 37 °C and pH 7.4 with an atmosphere of 5% CO_2_ in a moistened incubation chamber. Normal human colonic fibroblasts, CCD Co-18 obtained from American Type Culture Collection (ATCC^®^ CRL-1459™), were maintained in Eagle’s media supplemented with 1% streptomycin/penicilin, 1% 1-glutamine with 10% FBS at 37 °C and pH 7.4 in a moistened incubator (5% CO_2_).

### Magnetic resonance imaging (MRI) of SnFe_2_O_4_ aggregates

Owing to the intrinsic paramagnetic property of SnFe_2_O_4_, the test SnFe_2_O_4_ aggregates can be analyzed with MRI technology. The relaxavity of the MRI was acquired with a scanner (ClinScan 7 T MRI (70/30 USR, Bruker BioSpin, Germany). Phantom MRI was conducted for samples obtained at increasing concentrations of SnFe_2_O_4_ of 0~5 mg/mL and fixed in agarose gel for elucidation. The sequence of spin echo was utilized. The experimental parameters used for imaging were as follows: the *in vitro* T2 relaxivity of SnFe_2_O_4_-containing samples measured by curve fitting at an echo time (TE, 165.6 ms).

### Immunofluorescence of test cells

The examined cells were placed onto the cover slips (1.5 × 10^5^ cells/dish) and cultured with the SnFe_2_O_4_ aggregates (1 mmol/L, 0.2 mL) for 12 h. Before the staining study, the test cells were flushed 3 times with PBS. The DCFH-DA stock solution was formulated to the desire working concentration (80 μM) in physiological solution (PBS). Cells were then incubated with the DCFH-DA aqueous solution for half hour at 37 °C. To further visualize the amounts of catalase within cells, intracellularly endogenous catalase was stained and then detected by using an immunochemical staining technique. Cells were fixed in 4% paraformaldehyde (20–30 min) and permeabilized with Triton X-100 (0.1%, 15 min). Cells were first incubated with an anti-catalase primary antibody (Abcam, Cambridge, MA, USA) for 2 h at room temperature. The secondary antibody, Alexa Fluor®, was used to conjugate with primary antibody for another 1 h. Blocking serum (10%) was used for the blocking procedure. The test cells were also examined using IHC staining technology to identify the cellular lysosomes (LysoTracker®, Thermo Fisher Scientific, Lafayette, CO, USA). They were also further counterstained with DAPI (Sigma-Aldrich) for observing cell nuclei before being imaged with a confocal laser scanning microscope (CLSM).

### Hemolysis test

Briefly, after centrifugation (4 °C, 3500 rpm) to get precipitated intact red blood cells from rat blood, a 10-fold-volumal PBS was added to the red blood cells. Next, 0.3 mL of the above solution was mixed with 1.2 mL of different concentrations of the SnFe_2_O_4_ aggregates dispersed in PBS (DI water as positive control). The mixture was centrifuged (3000 rpm) and then the amount of released hemoglobin to the supernatant PBS was spectrophotometrically recorded with a microplate reader (500–640 nm).

### Cytotoxicity

Cells attached to a confocal dish were stained by with a Live/dead^®^ viability/cytotoxicity kits (Molecular Probes, Eugene, OR, USA). Ethidium homodimer (Eth-D) and acetoxymethylester of calcein, (calcein-AM) stock chemical compounds were adjusted to their final working concentrations in PBS as suggested by protocol. The samples were then incubated in these blends at room temperature for half hour. Fluorescent image were recorded by using a CLSM.

### Cell viability assay

Cells (at a density of 2 × 10^4^ cells/mL) were distributed into 96-well plates with cultured growth media in a moistened incubator at 37 °C with an atmosphere of 5% CO_2_ overnight for allowing cell attachment. Cell growth medium solution was then substituted by 200 μL of a SnFe_2_O_4_ aggregate suspension (1 mmol/L). After incubation for 12 h, the spent medium was withdrawn and newly cultured media (200 μL) including 20 μL of an MTT regaent (5 g/L in PBS) were added for further cultivation (within 4 h, 37 °C). Next removing the above cultured solution, the formazan reaction products were dissolved into a 150 μL of dimethylsulfoxide (DMSO) for reacting 20 min and then were extracted for microplate reader study. The absorbent optical density OD value at given 490 nm was detected by a microplate reader.

### Statistical analysis

ROS production and MTT experimental results were expressed as average ± standard deviation (SD). For comparing the means of group pairs, Student’s *t*-test was selected to analyse data. Differences result were considered as significant in case p < 0.05.
